# General Innovations in Pain Management

**DOI:** 10.3390/jcm14175957

**Published:** 2025-08-23

**Authors:** Alison Deng, Milan Patel, Cole Eigner, Robert Moghim, Alaa Abd-Elsayed

**Affiliations:** 1 Department of Medicine, Loyola University Chicago Stritch School of Medicine, Maywood, IL 60153, USA; adeng1@luc.edu; 2 Department of Medicine, New York Institute of Technology College of Osteopathic Medicine at Arkansas State University, Jonesboro, AR 72401, USA; mpate337@nyit.edu; 3 Department of Anesthesiology, University of Wisconsin School of Medicine and Public Health, Madison, WI 53726, USA; ceigner@wisc.edu; 4 Interventional Spine and Pain at Colorado Pain Care, Denver, CO 80222, USA; robert.moghim@coloradopaincare.com

**Keywords:** chronic pain, pain management, neuromodulation, regenerative medicine, pharmacogenetics, artificial intelligence, wearable health technology, integrative medicine

## Abstract

Chronic pain management is constantly evolving, and our literature review aims to describe the general innovations happening within the field. The need for advancements in chronic pain is a necessity, as debilitating back pain and other forms of chronic pain are significant issues in the United States. Traditionally, medications have been the initial treatment options in cases of chronic pain; however, the advancement in pharmacogenetics has led to an increased ability to create more personalized medication plans. Additionally, neuromodulation in spinal cord stimulation, transcranial magnetic stimulation, transcranial direct-current stimulation, and dorsal root ganglion stimulation continue to see increased usage in mainstream chronic pain management. These techniques have continued to prove successful in many chronic pain management cases. They are allowing practicing physicians more confidence in the variety of treatment options. Lastly, great strides have also been made in stem cell and regenerative therapies, such as platelet-rich plasma injections, and artificial intelligence, further advancing the various treatment options and overall efficiency of pain management. This review aims to critically analyze and review the most up-to-date literature within each section mentioned and comprehensively discuss the future of innovation in chronic pain management.

## 1. Introduction

Pain, defined by the International Association for the Study of Pain as “an unpleasant sensory and emotional experience,” is a prevalent and costly public health issue [1,2]. It affects a significant proportion of the United States (US). In fact, in a 2021 Centers for Disease Control and Prevention study, it was reported that 20.9% of US adults, or 51.6 million people, experienced chronic pain [3]. The experience of pain significantly impacts a person’s quality of life and interferes with one’s ability to accomplish daily living activities. One estimate found that the financial cost of pain in the US totaled USD 560–635 billion annually due to medical expenses and lost productivity [4]. These numbers are mirrored globally, with some studies estimating that more than 30% of the worldwide population is affected by chronic pain [5].

As pain significantly burdens individuals and society, medicine has placed considerable emphasis on developing treatments to relieve that burden. Pain management is the field of medicine that encompasses the treatment, therapies, and interventions that ease pain and improve a person’s ability to function. Personalized and appropriate treatment depends on the type of pain [6]. Pain is broadly divided into acute and chronic categories that characterize the duration of the pain. Acute pain lasts a shorter period, while chronic pain lasts three months or more. Pain types are further divided by the mechanism of action. There are three categories: nociceptive, neuropathic, and, more recently accepted, nociplastic [7]. All three can co-occur in an individual. Nociceptive pain arises from acute external stimuli such as trauma, chemical stimuli, and heat [8]. Pain is localized and may accompany visible injury. Second, neuropathic pain arises from neural damage such as diabetes, shingles, or neural injury. Finally, nociplastic pain is systemic due to augmented central nervous system pain and sensory processing. Symptoms include multifocal pain alongside typical pain co-symptoms of fatigue, altered sleep, mood disorders, and tenderness [7,9]. Nociplastic pain is not yet fully understood. Acknowledging all three mechanisms is critical to developing therapies that target each.

While understanding the mechanisms of pain is essential for developing effective treatments, it is equally important to recognize how approaches to pain relief have evolved over time. To understand the context of pain management today, we will briefly review the history of pain and medicine. The earliest evidence of pain management was in early civilizations of the Andean region, chewing coca leaves, which contain cocaine, a powerful and addictive painkiller, dating back over 3000 years [10]. With coca’s Euro-American expansion came concerns about its addictive properties in the early twentieth century. In the 1980s, pain was a growing concern and was recognized as an essential problem to treat [11]. In the United States, opioids were seen as potentially less addictive, setting a dangerous basis for the opioid epidemic to come.

The pharmaceutical industry, and specifically Purdue Pharma, aggressively marketed OxyContin for not only acute but also chronic pain [12]. By the mid-1990s, prescriptions soared. In reality, opioids are not suited for chronic pain as a first-line treatment in most cases, as individuals can develop tolerance; opioids can be used in instances of cancer-related pain and palliative care [13]. In parallel, nonsteroidal anti-inflammatory drugs (NSAIDs) were designed to treat inflammation, a precursor to pain [14]. The first NSAID, aspirin, was first added to the market in 1899 [15]. Although not addictive like opioids, NSAIDs have adverse side effects, including gastrointestinal damage and cardiovascular risks [16].

Furthermore, NSAIDs are not as suitable for severe pain. There is growing interest in seeking alternatives for pain treatment. Pain has traditionally been treated from a biomedical perspective with pharmacological medication. Holistic approaches can be challenging to implement due to complications with eligibility for insurance reimbursement, limiting access to treatments [16].

With the recognition of the addictive properties of opioids, the particular use cases for opiates, and the limitations of NSAIDs comes a call for innovation in the pain management field. There is an increasing focus on the comprehensive treatment of patients and the urge to discover upstream mechanisms and target these molecular and physiological pathways to personalize treatment [17].

Given the importance of understanding current therapies to develop novel treatments, this comprehensive review will provide an overview of novel pharmacological, interventional, and technological innovations that will shape the future of pain management. Relevant concepts include neuromodulation techniques, regenerative and cellular therapies, digital health and artificial intelligence, integrative and lifestyle medicine, and ethical considerations.

## 2. Pharmacological Innovations

Due to concerns over traditional pharmaceuticals for pain management, there have been studies exploring alternative non-opioid medications. For one, there is a growing body of interest surrounding the use of cannabinoids for pain relief. Several studies have found that cannabis use in patients with chronic pain can be effective [18,19,20]. However, there are criticisms surrounding the legal policies and the robustness of the research on cannabinoids [21]. Further clinical trials are required to solidify recommendations and update policies. However, the U.S. Food and Drug Administration has stringent regulations regarding the use of cannabis and its research [22].

Additionally, there is a basis of research that shows that antidepressants and anticonvulsants are effective in treating neuropathic pain, where the central nervous system is involved [23]. While they have been established as treatments, novel mechanisms of action can be exploited to develop more therapies. Acetyl-L-carnitine is an emerging neuropathic pain therapeutic that can promote neuronal plasticity and regulate neurotransmitters, including glutamate and gamma-aminobutyric acid (GABA) [24].

Aside from new mechanisms, there have been innovations in the delivery of drugs. Current treatments are systemic. Nanoparticles are an emerging technology that targets treatment and controlled release [25]. One particular type of nanoparticle, liposomal formulations are lipid vesicles that can transport drugs to target specific tissues. This method shows promise in precise delivery and fewer adverse side effects [26]. Modifying the lipid surface allows the vesicle to recognize a tissue or cell. Thus, drugs could even be encapsulated in vesicles that target receptors on the blood–brain barrier to get through the selective barrier. Opioids could be delivered to a local area to improve their pharmacokinetics and sustain pain relief while potentially preventing harmful respiratory depression side effects [26]. Exosomes are a type of small lipid formation secreted by cells. These exosomes have been tested to treat osteoarthritis, a painful joint disease characterized by chronic inflammation. Proteins in exosomes derived from mesenchymal stem cells and the placenta have anti-inflammatory, pro-proliferative properties that could be delivered to treat osteoarthritis in vivo. There is evidence to suggest that these exosomes could be used to treat other chronic pain conditions as well, including intervertebral disc disease, chemotherapy-induced pain, and cancer-induced pain [27]. Liposomal formulation technology unlocks limitless potential for future discoveries.

Furthermore, some nanodevices can detect the source of pain [25]. Quantum dot nanoparticles have optical properties that can quantify pain biomarkers based on unique fluoroscopic signatures. While biomarkers are still being discovered, there are established associations with matrix metalloproteinases in neuropathic pain and IL-6 in osteoarthritis [25]. Identifying the source of pain is essential for precise treatment targeting.

There is a new era of medicine with individualized treatments. Personalized medicine can be accomplished through pharmacogenetics, which analyzes how genetic variations affect drug effectiveness and tolerance for individuals. Several polymorphisms have been recognized as having clinical relevance for pain management. These recommendations may be dosage-based. More specifically, a single-nucleotide polymorphism in the opioid receptor mu one gene (*OPRM1*) 118 A>G leads to increased binding of endorphins [28]. Thus, these patients are proposed to have greater pain relief and require less morphine. Personalized recommendations based on a patient’s profile may also indicate a specific medication. Polymorphisms may lead to a reduced ability to metabolize a drug or be associated with adverse side effects. Opioids—including codeine, tramadol, and hydrocodone—and NSAIDs are metabolized by the hepatic cytochrome P450 2D6 (*CYP2D6*) enzyme [27]. Different polymorphisms lead to different abilities to metabolize opioids and NSAIDs. *CYP2D6* loss-of-function mutations can lead to poor metabolism that may leave individuals prone to GI bleeds from NSAIDs, respiratory depression from tramadol, or inability to achieve sufficient analgesia. On the other hand, rapid metabolizers may have toxic, high levels of opioids [28]. These outcomes are essential for developing a treatment plan and monitoring potential adverse side effects.

## 3. Neuromodulation Techniques

Neuromodulation techniques are electrical therapies to alter neuronal pathways, commonly associated with neuropathic pain and nociplastic pain [29]. These techniques include spinal cord stimulation (SCS), dorsal root ganglion (DRG) stimulation, peripheral nerve stimulation (PNS), vagal nerve stimulation (VNS), transcranial magnetic stimulation (TMS), and transcranial direct-current stimulation (tDCS).

SCS is a minimally invasive procedure that places electrodes near the spinal cord. Electrical stimulation suppresses pain perception in patients by activating inhibitory interneurons that release GABA. Until recently, SCS systems have remained open-looped despite needing manual adjustment due to electrode migration or intensity changing between standing and lying positions [30]. Changing the distance between the target tissue and the electrode delivers a different voltage. A recent breakthrough in developing a closed-loop feedback system allows the system to measure voltage changes in tissue fibers after an electrical pulse. Following the voltage reading, the system can adjust to deliver a consistent voltage [30]. A 2024 randomized clinical trial found that closed-loop SCS significantly reduced pain compared to open-loop SCS over 36 months [31]. At the 36-month mark of the study, more closed-loop (CS)-SCS patients, compared to open-loop (OL)-SCS, showed greater than or equal to approximately 50% pain reduction. This randomized controlled trial (RCT) further supports the great usage of spinal cord stimulation, specifically CL-SCS, in treating chronic back and leg pain [31].

Additionally, a decade ago, the Federal Drug Administration approved high-frequency SCS. Typically, SCS is conducted at frequencies around 50–60 Hz, while high-frequency SCS uses higher frequencies at 10 kHz. While traditional SCS causes paresthesia and tingling to mask pain, high-frequency SCS does not. Studies have shown that high-frequency SCS significantly reduces pain compared to typical SCS [32,33]. These innovations in SCS have improved the practicality and effectiveness of pain treatments.

Furthermore, there is a promising synergistic effect between upcoming technology and SCS. Artificial intelligence (AI) can revolutionize the personalized medicine approach to neuromodulation. This technology can be trained to learn an individual’s movement patterns and biomarkers. For example, applying AI to SCS approaches can more efficiently and accurately make delivery adjustments in pulse frequency, intervals, and amplitude based on movement patterns and physiological signs. Furthermore, AI creates a more efficient model with predictive capabilities to recommend treatment based on the patient’s pain level, location, comorbidities, and prior treatment responses, rather than having the patient test various technologies to find which is suitable [34,35,36,37,38,39].

Minimally invasive DRG stimulation is a therapy of increasing interest, with promising results. It is indicated in cases of chronic, localized, neuropathic pain. It is especially beneficial in lower-extremity pain, including hip, knee, groin, and foot neuropathy, whereas traditional SCS is not targeted [40]. Several observational studies have demonstrated the efficacy of DRG stimulation in lower-extremity neuropathy. Across one RCT and eleven observational studies, DRG stimulation was seen as the superior treatment method in comparison to traditional dorsal column SCS at a 12-month time point. Furthermore, approximately 74% of patients achieved the primary endpoint of success at the 12-month mark in the DRG group, as compared to the 53% in the SCS cohort [40]. However, there were small patient populations in many of these studies. Current evidence is limited, warranting more studies into the effectiveness of DRG stimulation.

PNS and VNS can be minimally invasive or non-invasive therapies. The availability of invasive and non-invasive techniques is instrumental in identifying and testing the treatment. For PNS, a non-invasive trial system can help indicate whether a permanent, invasive PNS therapy is the proper method to help alleviate pain for patients [41]. There have also been innovations in the devices used for PNS, including the Nalu Neurostimulation System, which is placed on the surface and can provide relief from chronic, neuropathic pain [42]. Additionally, one innovation in PNS placement through percutaneous leads with ultrasound guidance has been found to produce safer placement and fewer complications. This placement can be completed in an ambulatory setting and includes features such as hardware miniaturization and the potential for using external implantable pulse generators [43]. For VNS, there are currently multiple studies on invasive and non-invasive technologies to relieve pain. These devices modulate the vagus nerve, which sends signals to brain areas related to pain processing [44]. VNS was first approved for use in managing epilepsy. Now, there are novel approaches to incorporating VNS in pain management.

TMS and tDCS are non-invasive, wearable devices that are being explored as potential treatments for pain relief. While both deliver current to the brain, TMS uses magnetic fields to generate electric currents, while tDCS applies an electrical current. There has been some evidence that TMS at the primary motor cortex (M1) may be effective for alleviating neuropathic pain, fibromyalgia, and migraines [45]. However, the response is not observed in all patients receiving TMS treatment. Thus, there is a need for further research to understand the limitations of TMS and the mitigating factors involved in pain response. Additionally, individual responses introduce a level of variability. tDCS was first explored for treating neurological and psychiatric disorders. Currently, there is an interest in applying tDCS to pain management. Of interest for future studies is the potential for tDCS to increase the pain tolerance of individuals by applying the device before pain arises [46]. Furthermore, there is evidence to support the notion that tDCS can provide chronic pain relief and can decrease the use of opioids after surgery [47]. Altogether, there is much promise on the horizon for neuromodulation techniques as alternative therapies for pain management.

Despite promising results, patients face enormous barriers in gaining access to these neuromodulation treatments. Particularly, these barriers can be socioeconomic and related to insurance type. One study found that patients covered under Medicare were 15% less likely to receive permanent SCS placement compared to those with private insurance; patients covered under Medicaid were 22% less likely to receive permanent placement [48]. Permanent placement also depends on geographical area, as providers need training and specialized equipment [49].

## 4. Regenerative and Cellular Therapies

Regenerative medicine has revolutionized its approach to chronic pain management by shifting from symptom-based management to cellular and tissue restoration. Among new strategies, stem cell therapy, platelet-rich plasma (PRP), and gene therapy have all emerged with the potential to modulate pain and, in some cases, reverse tissue degeneration. These new applications are relevant to diseases such as osteoarthritis and discogenic lower-back pain, as current assessment and treatment commonly miss underlying causes.

Osteoarthritis is an irreversible degeneration of the synovial joint, the fluid-filled knee cavity that allows for a wide range of motion. The condition affects the daily activities of nearly every individual diagnosed [50]. These new techniques have been introduced to decrease degeneration and prevent patient pain. A total of 1093 patients affected by either bilateral or unilateral osteoarthritis were taken into 23 studies and given either PRP or mesenchymal stem cells (MSCs). PRP is a blood-derived product with a high concentration of plasma that can deliver high amounts of growth factor, while mesenchymal stem cells or MSCs are heterogeneous stem cells isolated from adult tissue, with the ability to differentiate and interact with many kinds of cells [50,51]. In the studies, patients were injected in the synovial joint with either the PRP, MSCs, or a combination of both, and then periodically monitored and measured using the Western Ontario and McMaster Universities Arthritis Index (WOMAC). The study found that both treatments were tolerated and effective in reducing the reported pain of individuals, alongside a measured decrease in cartilage loss in a few MSC trials. Other studies have found that PRP is potentially more effective than the current modulation using hyaluronic acid (HA) injections to the knee joint [51]. This outcome is supported by a 2019 meta-analysis of fifteen randomized controlled trials encompassing 1314 patients, which found that PRP injections were more effective than HA for reducing pain related to knee osteoarthritis at 6 and 12 months [52]. Furthermore, the study observed improved functionality for patients receiving PRP [52]. While PRP is more effective, it is not as often covered by insurance, and it is more expensive. PRP and stem cell injections have also been studied to treat sacroiliac joint dysfunction. The sacroiliac joint is a large axial spine and pelvis joint, with a critical role in weight transfer between the upper and lower body. The sacroiliac joint can be a source of lower-back and lower-extremity pain. PRP and stem cells are also considered for treating facet joint-related axial spine pain. Currently, limited evidence supports the effectiveness of PRP and stem cell treatments in the sacroiliac and facet joints. However, these treatments are gaining support for other joints [51,53].

Discogenic back pain originates from the degeneration or damage of one or multiple intervertebral discs in the spine. This damage can similarly disrupt patients’ daily lives and can cause persistent pain. Numerous studies have been compiled and reviewed regarding the nature and similarity of stem cell therapy. The review involving stem cell therapy injection of MSCs provides promising data from studies carried out involving the injection of MSCs into the damaged tissue of the spine, suggesting that further non-surgical techniques can be used in treatment despite isolation and host delivery [54]. Additionally, studies have been performed using PRP as a potential candidate for promoting disc regeneration. The procedure similarly involves the injection of PRP into the degenerate tissue. The blood concentrate can release high concentrations of particles and facilitate the release of healing growth factors to damaged tissue. The comprehensive review of studies suggests that PRP is highly effective in stimulating IVD tissue to repair degenerated discs [55].

## 5. Digital Health and Artificial Intelligence in Pain Management

Technological advancements in how chronic pain is monitored and assessed are sources of interest for the future of pain management. Wearable devices are an increasingly common approach to incorporating digital health in the field. One study focused on assessing iPhone and Apple Watch applications to monitor movement, sleep, and self-reported pain scores. Over 12 months, 105 patients with chronic pain were given wearable health technology (WHT), 146 patients in the same program were not given WHT, and 161 patients remained on their medication regimen only, averaging 143 days of WHT usage across the study. Across the control and experimental groups, there was a decrease in depression scores as well as morphine usage. While there were positive results in both groups, the results are statistically significant; thus, further research needs to be conducted to confirm these initial results [56].

Wearable devices and digital health have also been linked to promoting physical activity amongst individuals with chronic pain. However, this area of chronic pain management is a growing field, with limited research available. Within the current view of this burgeoning field, wearable technology has shown an initial positive correlation between promoting physical activity through physiological signals and reducing adverse outcomes. Specifically, greater attention and future research should be directed towards assessing physical activity and capabilities related to the physiological signals produced, as well as how pain in the individual is altered [50,51].

In addition to wearable technology, AI has become increasingly involved in medicine. AI is currently applied as a diagnostic aid for clinicians and as a tool to model pain progression and predict individual treatment responses. Researchers have attempted to quantify pain through analyzing physiological data, with some success [51]. AI will seek to optimize and improve four main areas within chronic pain management: outcome prediction based on clinical information, information extraction from patient notes, modeling omics data for patient subgroup identification, and disentangling complex neuronal pain signals [52,56,57].

Beginning with optimizing outcome prediction and clinical information, the movement to large-scale electronic health record (EHR) systems has allowed much patient information to be centralized in one place. Information, including patient imaging, laboratory results, pre- and post-op results, and more, can be analyzed through machine learning (ML) algorithms to provide advanced clinical outcomes and next steps [58,59]. AI has already shown promising results in aiding physicians using cognitive behavioral therapy (CBT) for chronic pain. In one study, an artificial intelligence-driven CBT treatment option had enough information approximately 94% of the time and could gradually evolve in its decision-making. As a result, positive outcomes reflected as “reward scores” increased amongst the study population [60].

## 6. Integrative and Lifestyle-Based Innovations

Recently, there has been growing interest in the role of lifestyle-oriented approaches to chronic pain management, beyond traditional medication. Integrative medicine combines conventional care with complementary therapies, which are used in combination with biomedical approaches rather than replacing conventional approaches (i.e., alternative therapies), pre- and post-operation to increase the effectiveness of pain management [61]. New approaches offer promising results in addressing the complex dimensions of pain in surgical care. Three notable approaches involve mind–body intervention, physical therapy, and nutritional intervention. When used as an additive therapy, these complementary approaches can holistically enhance a treatment plan.

Mind–body interventions include therapies such as meditation, CBT, and biofeedback, designed to enhance the mind’s ability to control the body and, in some cases, alleviate pain. Meditation in medicine can be described by the body’s ability to focus and redirect thoughts away from negative feelings and sensations [62]. A study was conducted that gathered a large number of patients who were either involved or uninvolved with meditation and also suffered from similar forms of chronic pain. The study analyzed patients involved in meditation alongside conventional care and showed that many patients involved in meditation reported lower pain. The studies suggest that some patients can further alleviate pain and stress by redirecting their thoughts to control the sensations. Cognitive behavioral therapy (CBT) and biofeedback are also techniques used within the scope of mind–body intervention [63]. CBT is a technique developed to help patients understand how their thoughts can influence their reaction to pain. At the same time, biofeedback aims to help patients become aware of and control bodily functions typically thought to be outside of conscious control. Studies were conducted implementing both techniques in a large group of patients experiencing chronic pain. The studies suggest that CBT is effective in aiding pain treatment, while biofeedback needs further investigation to prove its effectiveness.

A wide variety of physical therapy techniques have also been developed to prevent pain and complications in patients before an operation. A study of patients undergoing cardiac and abdominal surgery required the patients to perform inspiratory muscle training (the muscles that aid in breathing) before their operations [64]. The patients were then monitored and assessed post-operation. The results indicated that patients involved in the physical therapy had shorter hospital stays post-operation and were less likely to have complications after or during surgery. Although more research is needed to correlate these findings to physical therapy directly, the findings suggest promising preventative care.

Nutritional interventions have been implemented to optimize a patient’s status and reduce pain before, during, and after surgical care. Two key components to nutritional intervention include anti-inflammatory diets and nutraceuticals—food components that go beyond basic nutrition, including omega-3 fatty acids, minerals, and vitamins. In a study, 46 patients for elective surgery were chosen to drink 1 L of enriched formula for 5 days preoperatively and 7 days postoperatively [65]. The results indicated that postoperative stay in the ICU was shorter in the enriched group than in the control, indicating that their pain was alleviated to a higher degree, and suggesting that nutritional intervention can aid in surgical care. A different study looked into gastrointestinal patients undergoing surgery [66]. The patients were administered nutraceuticals before their operations as a protein supplementation high in omega fatty acids against a placebo group. The results indicated that patients given nutraceuticals were less likely to have postoperative complications and pain.

## 7. Challenges and Ethical Considerations

As the scope of pain management evolves due to advances in technology and breakthroughs in science, concerns are raised regarding the ethical use of AI technology and the disparities present in populations regarding race, socioeconomic status, and location. These concerns continue to be explored to ensure that all patients receive ethical care in pain management.

The integration of AI technology in pain management provides the opportunity for enhanced patient monitoring and care, with potential ethical issues regarding transparency and data privacy. A study used a learning-based predictive AI model for identifying the risk of persistent opioid use post-operation [67]. The system offers the promising ability to detect those susceptible to opioid addiction and give physicians a heads-up to the potential issue before it becomes a reality. Despite this, there are still speculations about the system’s data collection and reliability. The recentness of AI’s explosion suggests that further research remains to be done on its effectiveness in the scope of healthcare.

Additionally, as technology continues to evolve in the field, further research has been carried out and is continuing to be conducted to address disparities in the field of pain management. A meta-analysis of a vast population was performed to reveal disparities within opioid prescription based on race [68]. The meta-analysis included a systematic search of prescription publications between 2011 and 2021 from the Scopus database. The results of the analysis indicated that both Black and Hispanic patients were less likely than White patients to be prescribed opioids for pain, despite similar self-reports of pain. This disparity remained consistent despite the decade gap between the first and last publications analyzed. These results suggest that much work still needs to be carried out to ensure equal and adequate care for every patient based on race.

## 8. Future Directions

Current research on cannabinoid use for pain management is limited in scope. Studies that recommend cannabis use are often limited to systematic reviews and meta-analyses. Many clinical trials have limitations, including small sample sizes, lack of longitudinal follow-up, standard care control groups, and lack of functional outcome results [23]. These are all components that need to be considered for a robust clinical trial to confirm cannabis use for pain management. Additionally, there needs to be a review of the current guidelines for physicians and the national policies for medical cannabis. Current policies place restrictions on the scope of research studies and clinical practice.

Furthermore, there is a need for more testing of the promising neuromodulation techniques on the horizon. In particular, randomized controlled trials of DRG are needed to determine its effectiveness and add to the current body of observational studies [34]. For new technology uses, such as determining the need for invasive PNS following non-invasive testing, follow-up with patients is required to determine whether the method effectively determines the correct course of action [33]. TMS is another technique that requires future studies to determine what factors precipitate the therapeutic inefficacy in some individuals [40].

There are other emerging areas of research with great potential. One of these is optogenetics, which delivers light to tissue and acts on light-sensitive proteins to control or visualize cell activity [69]. Optogenetics is a field where translational bench-to-bedside practices would be beneficial in elucidating the molecular pathways behind neuropathic pain and developing therapeutics to treat molecular targets. One study successfully prevented pain by stimulating mice’s halorhodopsin, a light-driven chloride transporter [69]. There is potential for discovering new treatments. Another study found that stimulating GABAergic lateral parabrachial nucleus neurons relieves pain-like behavior in vivo [70,71]. This method can help to examine neuronal pathways involved in the pain response. Optogenetics studies are currently restricted to animal models only. The transition to clinical settings will come with unique challenges in complex systems. Of note, the transition from animal to human studies will present major difficulties due to physiological differences between the species and additional regulation and recruitment challenges for clinical studies.

Neuroimmune interactions are of great interest for the immune system’s role in initiating and maintaining chronic pain. Due to the immune system’s critical role, it is a potential target for therapeutics. While there are many therapeutics for managing inflammatory pain, including rheumatoid arthritis, there is interest in developing more therapeutics for different conditions [72]. A study that tested a chemokine receptor 2 (CCR2) antagonist found that it was ineffective in relieving pain from posttraumatic neuralgia (nerve pain after trauma) compared to the placebo [73]. The antagonist interacted with CCR2 and had no safety or tolerability issues. CCR2 has been found to mediate chronic pain [74,75]. Thus, it is an essential target of potential therapeutics. While this study focused on posttraumatic neuralgia, it has the potential to be tested for other conditions.

Multimodal interdisciplinary approaches are increasingly of interest and importance. A holistic approach includes physical or occupational therapy and behavioral therapy. Biofeedback is a behavioral therapy that requires additional studies into its long-term efficacy, determining its use cases, and conducting a large-scale randomized controlled trial [76,77]. Additionally, research on integrating pharmaceuticals, physical therapy, and behavioral therapy has not been based on standardized doses and treatments [78]. Without the standardization of treatment, it is not easy to draw conclusions and create a protocol for clinical practice. A standard protocol should be developed in the future, and all dose and time procedures should be reported.

With the advent of AI comes its many potential and beneficial uses in medicine. Notably, remote patient monitoring with wearable technology, in combination with AI, can assist in managing chronic conditions, including chronic pain. Remote patient monitoring can provide and transmit real-time data, allowing for more informed decisions. AI can enhance one’s quality of life through personalized recommendations. Several challenges persist in ensuring the accuracy of data that AI is trained on, including the fact that the data should be free of biases. The generalizability of AI models is particularly important to ensure that they do not exacerbate existing inequities or recommend inaccurate information. Furthermore, as treatment strategies constantly evolve, AI must incorporate these new findings [54].

## 9. Conclusions

With the current limitations and concerns around traditional pharmaceuticals for pain management, there have been other options that have gained popularity. Cannabinoids are one example in which there have been great initial results in the field of chronic pain management. Additionally, antidepressants and anticonvulsants have also been effective options for neuropathic pain in particular. There has been continued research in this field, such as acetyl-L-carnitine as an agent of interest through its ability to modulate neurotransmitters and neuronal plasticity. Furthermore, drug delivery innovations, including liposomes and nanoparticles, are changing how medications are being used to reach target tissues.

The increased usage of pharmacogenetics in clinical medicine has also shown a prioritization of personal care. Genetic polymorphisms such as the *OPRM1* gene and CYP2D6 influence a patient’s response to opioids and NSAIDs. Thus, this has implications for treatment options’ overall effects and safety. However, using tailored treatments based on the genetic profiles is how chronic pain management is progressing.

Neuromodulation is a growing field in pain management. This field offers non-opioid alternatives via electrical stimulation of neural pathways. These options are becoming increasingly popular, while the development of high-frequency and closed-loop feedback systems has steadily improved outcomes. The ability to adjust stimulation in real time has allowed the ability to address electrode migration and postural shifts.

Non-invasive brain stimulation techniques are growing in use and application within pain management. Particularly, TMS has shown potential as a treatment option for fibromyalgia, migraines, and neuropathic pain. On the other hand, tDCS may reduce postoperative opioid use and enhance potential pain tolerance in preventative settings.

Regenerative medicine has led to a shift in pain management, focusing on cellular repair and tissue regeneration. Stem cell treatment and platelet-rich plasma have shown potential in reversing tissue degeneration in conditions like osteoarthritis and discogenic back pain. In the key study used in this section, the patients showed positive results from treatment. Together, these two options, as alternatives to surgical approaches, are significant advancements in pain management that further pain management and the regeneration of functional capability.

Innovations through technological advancements have also helped further progress the field of pain management. Tools such as WHT and AI are leading the way in pain management treatment plans. Smartwatches and mobile applications are examples of wearables that can help monitor patient information in real time. In addition to monitoring, these devices have shown the ability to help patients become more active and detect physiological signals indicative of changes in pain levels. Thus, with this information, new avenues of treatment plans, in the form of tailored changes and personalized feedback, are more viable than ever.

AI is also emerging as a great tool in pain management. Its ability to aid in outcome prediction using electronic health records, model omics data for patient subgroup identification, and create personalized treatment plans has raised the ceiling of care available to patients. AI has also been able to help physicians extract information from clinical notes and increase efficiency. Cognitive behavioral therapy has also demonstrated promising results and improved patient outcomes. Advancements in digital health and machine learning have led to great potential for growth in pain management and more precise treatment plans in chronic pain.

## Data Availability

Not applicable.

## References

[1] Raja S.N., Carr D.B., Cohen M., Finnerup N.B., Flor H., Gibson S., Keefe F.J., Mogil J.S., Ringkamp M., Sluka K.A. (2020). The revised International Association for the Study of Pain definition of pain: Concepts, challenges, and compromises. Pain.

[2] Katzman J.G., Gallagher R.M. (2024). Pain: The Silent Public Health Epidemic. J. Prim. Care Community Health.

[3] Rikard S.M., Strahan A.E., Schmit K.M., Guy G.P. (2023). Chronic Pain Among Adults—United States, 2019–2021. MMWR Morb. Mortal. Wkly. Rep..

[4] Gaskin D.J., Richard P. (2011). Appendix C The Economic Costs of Pain in the United States. Relieving Pain in America: A Blueprint for Transforming Prevention, Care, Education, and Research.

[5] Cohen S.P., Vase L., Hooten W.M. (2021). Chronic pain: An update on burden, best practices, and new advances. Lancet.

[6] Nalamachu S. (2013). An overview of pain management: The clinical efficacy and value of treatment. Am. J. Manag. Care.

[7] Fitzcharles M.-A., Cohen S.P., Clauw D.J., Littlejohn G., Usui C., Häuser W. (2021). Nociplastic pain: Towards an understanding of prevalent pain conditions. Lancet.

[8] Armstrong S.A., Herr M.J. (2025). Physiology, Nociception. StatPearls.

[9] Kaplan C.M., Kelleher E., Irani A., Schrepf A., Clauw D.J., Harte S.E. (2024). Deciphering nociplastic pain: Clinical features, risk factors and potential mechanisms. Nat. Rev. Neurol..

[10] Biondich A.S., Joslin J.D. (2016). Coca: The History and Medical Significance of an Ancient Andean Tradition. Emerg. Med. Int..

[11] DeWeerdt S. (2019). Tracing the US opioid crisis to its roots. Nature.

[12] Omoigui S. (2007). The biochemical origin of pain: The origin of all pain is inflammation and the inflammatory response. Part 2 of 3—Inflammatory profile of pain syndromes. Med. Hypotheses.

[13] Horn D.B., Vu L., Porter B.R., Afzal M. (2025). Responsible Controlled Substance and Opioid Prescribing. StatPearls [Internet].

[14] Rao P., Knaus E.E. (2008). Evolution of nonsteroidal anti-inflammatory drugs (NSAIDs): Cyclooxygenase (COX) inhibition and beyond. J. Pharm. Pharm. Sci..

[15] Payne R. (2000). Limitations of NSAIDs for pain management: Toxicity or lack of efficacy?. J. Pain.

[16] Schatman M.E. (2011). The role of the health insurance industry in perpetuating suboptimal pain management. Pain Med..

[17] Slitzky M., Yong R.J., Lo Bianco G., Emerick T., Schatman M.E., Robinson C.L. (2024). The Future of Pain Medicine: Emerging Technologies, Treatments, and Education. J. Pain Res..

[18] Hill K.P. (2015). Medical Marijuana for Treatment of Chronic Pain and Other Medical and Psychiatric Problems: A Clinical Review. JAMA.

[19] Degenhardt L., Lintzeris N., Campbell G., Bruno R., Cohen M., Farrell M., Hall W.D. (2015). Experience of adjunctive cannabis use for chronic non-cancer pain: Findings from the Pain and Opioids IN Treatment (POINT) study. Drug Alcohol Depend..

[20] Lynch M.E., Campbell F. (2011). Cannabinoids for treatment of chronic non-cancer pain; A systematic review of randomized trials. Br. J. Clin. Pharmacol..

[21] Mallick-Searle T., Marie B.S. (2019). Cannabinoids in Pain Treatment: An Overview. Pain Manag. Nurs..

[22] U.S. Food and Drug Administration FDA and Cannabis: Research and Drug Approval Process. https://www.fda.gov/news-events/public-health-focus/fda-and-cannabis-research-and-drug-approval-process#.

[23] Sullivan M.D., Robinson J.P. (2006). Antidepressant and anticonvulsant medication for chronic pain. Phys. Med. Rehabil. Clin. N. Am..

[24] Bernatoniene J., Sciupokas A., Kopustinskiene D.M., Petrikonis K. (2023). Novel Drug Targets and Emerging Pharmacotherapies in Neuropathic Pain. Pharmaceutics.

[25] Bhansali D., Teng S.L., Lee C.S., Schmidt B.L., Bunnett N.W., Leong K.W. (2021). Nanotechnology for Pain Management: Current and Future Therapeutic Interventions. Nano Today.

[26] Martinez J., Ingram N., Kapur N., Jayne D.G., Beales P.A. (2024). Vesicle-based formulations for pain treatment: A narrative review. Pain Rep..

[27] Shipman W.D., Fonseca R., Dominguez M., Bhayani S., Gilligan C., Diwan S., Rosenblum D., Ashina S., Tolba R., Abd-Elsayed A. (2024). An Update on Emerging Regenerative Medicine Applications: The Use of Extracellular Vesicles and Exosomes for the Management of Chronic Pain. Curr. Pain Headache Rep..

[28] Kaye A.D., Garcia A.J., Hall O.M., Jeha G.M., Cramer K.D., Granier A.L., Kallurkar A., Cornett E.M., Urman R.D. (2019). Update on the pharmacogenomics of pain management. Pharmacogenomics Pers. Med..

[29] Knotkova H., Hamani C., Sivanesan E., Le Beuffe M.F.E., Moon J.Y., Cohen S.P., Huntoon M.A. (2021). Neuromodulation for chronic pain. Lancet.

[30] Mangano N., Torpey A., Devitt C., Wen G.A., Doh C., Gupta A. (2025). Closed-Loop Spinal Cord Stimulation in Chronic Pain Management: Mechanisms, Clinical Evidence, and Emerging Perspectives. Biomedicines.

[31] Mekhail N.A., Levy R.M., Deer T.R., Kapural L., Li S., Amirdelfan K., Pope J.E., Hunter C.W., Rosen S.M., Costandi S.J. (2024). ECAP-controlled closed-loop versus open-loop SCS for the treatment of chronic pain: 36-month results of the EVOKE blinded randomized clinical trial. Reg. Anesth. Pain Med..

[32] Torres-Bayona S., Mattar S., Arce-Martinez M.P., Miranda-Acosta Y., Guillen-Burgos H.F., Maloof D., Samprón N., Farah J.-O. (2021). High frequency spinal cord stimulation for chronic back and leg pain. Interdiscip. Neurosurg..

[33] Uccelli A., Moretta L., Pistoia V. (2008). Mesenchymal stem cells in health and disease. Nat. Rev. Immunol..

[34] Kataria S., Inggas M.A.M., Patel U., Wijaya J.H., Yabut K., Ayub M.A., Maniyar P., Upadhyay N., Davitashvili B., Patel J. (2025). A Systematic Review and Meta-Analysis of Stem Cell Therapies for Pain in Diabetic Neuropathy, Osteoarthritis, and Spinal Cord Injuries. Curr. Pain Headache Rep..

[35] Barakat A.H., Elwell V.A., Lam K.S. (2019). Stem cell therapy in discogenic back pain. J. Spine Surg..

[36] Abd-Elsayed A., Robinson C.L., Marshall Z., Diwan S., Peters T. (2024). Applications of Artificial Intelligence in Pain Medicine. Curr. Pain Headache Rep..

[37] Chang Y., Yang M., Ke S., Zhang Y., Xu G., Li Z. (2020). Effect of Platelet-Rich Plasma on Intervertebral Disc Degeneration In Vivo and In Vitro: A Critical Review. Oxidative Med. Cell. Longev..

[38] Prunskis J.V., Masys T., Pyles S.T., Abd-Elsayed A., Deer T.R., Beall D.P., Gheith R., Patel S., Sayed D., Moten H. (2025). The Application of Artificial Intelligence to Enhance Spinal Cord Stimulation Efficacy for Chronic Pain Management: Current Evidence and Future Directions. Curr. Pain Headache Rep..

[39] Abraham M.E., Gold J., Dondapati A., Sheaffer K., Gendreau J.L., Mammis A. (2021). High Frequency 10 kHz Spinal Cord Stimulation as a First Line Programming Option for Patients with Chronic Pain: A Retrospective Study and Review of the Current Evidence. Cureus.

[40] D’Souza R.S., Kubrova E., Her Y.F., Barman R.A., Smith B.J., Alvarez G.M., West T.E., Abd-Elsayed A. (2022). Dorsal Root Ganglion Stimulation for Lower Extremity Neuropathic Pain Syndromes: An Evidence-Based Literature Review. Adv. Ther..

[41] Yalamuru B., Nia S. (2024). Peripheral nerve stimulation on trial: A novel, cost-effective approach to determine patient candidacy prior to implantation. Interv. Pain Med..

[42] Kalia H., Pritzlaff S., Li A.H., Ottestad E., Gulati A., Makous J., Chakravarthy K. (2022). Application of the novel Nalu™ Neurostimulation System for peripheral nerve stimulation. Pain Manag..

[43] Abd-Elsayed A., D’Souza R.S. (2021). Peripheral Nerve Stimulation: The Evolution in Pain Medicine. Biomedicines.

[44] Shao P., Li H., Jiang J., Guan Y., Chen X., Wang Y. (2023). Role of Vagus Nerve Stimulation in the Treatment of Chronic Pain. Neuroimmunomodulation.

[45] Fernandes A.M., Graven-Nielsen T., de Andrade D.C. (2022). New updates on transcranial magnetic stimulation in chronic pain. Curr. Opin. Support. Palliat. Care.

[46] Fregni F., Macedo I.C., Spezia-Adachi L.N., Scarabelot V.L., Laste G., Sanchesf P.R.S., Caumo W., Torres I.L.S. (2018). Transcranial direct current stimulation (tDCS) prevents chronic stress-induced hyperalgesia in rats. Brain Stimul..

[47] Pacheco-Barrios K., Cardenas-Rojas A., Thibaut A., Costa B., Ferreira I., Caumo W., Fregni F. (2020). Methods and strategies of tDCS for the treatment of pain: Current status and future directions. Expert Rev. Med. Devices.

[48] Day W., Maloy G.C., Winter A.D., Chapman K.B., Seddio A.E., Doshi R.H., Ratnasamy P.P., Varthi A.G., Fourman M.S., Grauer J.N. (2025). Spinal cord stimulator utilization trends and predictors of unsuccessful trial-to-implant conversion. N. Am. Spine Soc. J..

[49] Huang K.T., Martin J., Marky A., Chagoya G., Hatef J., Hazzard M.A., Thomas S.M., Lokhnygina Y., Lad S.P. (2015). A national survey of spinal cord stimulation trial-to-permanent conversion rates. Neuromodulation J. Int. Neuromodulation Soc..

[50] Ip H.L., Martin J., Marky A., Chagoya G., Hatef J., Hazzard M.A., Thomas S.M., Lokhnygina Y., Lad S.P. (2020). Regenerative Medicine for Knee Osteoarthritis—The Efficacy and Safety of Intra-Articular Platelet-Rich Plasma and Mesenchymal Stem Cells Injections: A Literature Review. Cureus.

[51] Redler L.H., Thompson S.A., Hsu S.H., Ahmad C.S., Levine W.N. (2011). Platelet-rich plasma therapy: A systematic literature review and evidence for clinical use. Physician Sportsmed..

[52] Han Y., Huang H., Pan J., Lin J., Zeng L., Liang G., Yang W., Liu J. (2019). Meta-analysis Comparing Platelet-Rich Plasma vs Hyaluronic Acid Injection in Patients with Knee Osteoarthritis. Pain Med..

[53] Manchikanti L., Kaye A.D., Abd-Elsayed A., Sanapati M.R., Pampati V., Shekoohi S., Hirsch J.A. (2025). A Systematic Review of Sacroiliac Joint Injections of Platelet-Rich Plasma (Prp) and Stem Cells. Curr. Pain Headache Rep..

[54] Manchikanti L., Abd-Elsayed A., Kaye A.D., Sanapati M.R., Pampati V., Shekoohi S., Hirsch J.A. (2025). A Systematic Review of Regenerative Medicine Therapies for Axial Spine Pain of Facet Joint Origin. Curr. Pain Headache Rep..

[55] Patel P.M., Green M., Tram J., Wang E., Murphy M.Z., Abd-Elsayed A., Chakravarthy K. (2024). Beyond the Pain Management Clinic: The Role of AI-Integrated Remote Patient Monitoring in Chronic Disease Management—A Narrative Review. J. Pain Res..

[56] Han J.J., Graham J.H., Snyder D.I., Alfieri T. (2022). Long-term Use of Wearable Health Technology by Chronic Pain Patients. Clin. J. Pain.

[57] Leroux A., Rzasa-Lynn R., Crainiceanu C., Sharma T. (2021). Wearable Devices: Current Status and Opportunities in Pain Assessment and Management. Digit. Biomark..

[58] Eboreime K.O., Hughes J.G., Lee R., Luo J. (2025). Can Wearable Device Promote Physical Activity and Reduce Pain in People with Chronic Musculoskeletal Conditions?. J. Clin. Med..

[59] Meier T.A., Refahi M.S., Hearne G., Restifo D.S., Munoz-Acuna R., Rosen G.L., Woloszynek S. (2024). The Role and Applications of Artificial Intelligence in the Treatment of Chronic Pain. Curr. Pain Headache Rep..

[60] Hagedorn J.M., George T.K., Aiyer R., Schmidt K., Halamka J., D’Souza R.S. (2024). Artificial Intelligence and Pain Medicine: An Introduction. J. Pain Res..

[61] Piette J., Newman S., Krein S.L., Marinec N., Chen J., Williams D.A., Edmond S.N., Driscoll M., LaChappelle K.M., Maly M. (2022). Artificial Intelligence (AI) to Improve Chronic Pain Care: Evidence of AI Learning. Intell.-Based Med..

[62] Hakami N. (2024). Integrating complementary and alternative medicine in surgical care: A narrative review. Medicine.

[63] Rajjoub R., El Sammak S., Rajjo T., Rajjoub N.S., Hasan B., Saadi S., Kanaan A., Bydon M. (2024). Meditation for perioperative pain and anxiety: A systematic review. Brain Behav..

[64] Wie C., Dunn T., Sperry J., Strand N., Dawodu A., Freeman J., Covington S., Pew S., Misra L., Maloney J. (2025). Cognitive Behavioral Therapy and Biofeedback. Curr. Pain Headache Rep..

[65] Valkenet K., van de Port I.G.L., Dronkers J.J., de Vries W.R., Lindeman E., Backx F.J.G. (2011). The effects of preoperative exercise therapy on postoperative outcome: A systematic review. Clin. Rehabil..

[66] Giger U., Büchler M., Farhadi J., Berger D., Hüsler J., Schneider H., Krähenbühl S., Krähenbühl L. (2007). Preoperative immunonutrition suppresses perioperative inflammatory response in patients with major abdominal surgery—A randomized controlled pilot study. Ann. Surg. Oncol..

[67] Serrano P.E., Parpia S., Simunovic M., Duceppe E., Pinto-Sanchez M.I., Bhandari M., Levine M. (2022). Perioperative optimization with nutritional supplements in patients undergoing gastrointestinal surgery for cancer: A randomized, placebo-controlled feasibility clinical trial. Surgery.

[68] Gabriel R.A., Park B.H., Hsu C.-N., Macias A.A. (2025). A Review of Leveraging Artificial Intelligence to Predict Persistent Postoperative Opioid Use and Opioid Use Disorder and its Ethical Considerations. Curr. Pain Headache Rep..

[69] Hirani S., Benkli B., Odonkor C.A., Hirani Z.A., Oso T., Bohacek S., Wiedrick J., Hildebrand A., Osuagwu U., Orhurhu V. (2024). Racial Disparities in Opioid Prescribing in the United States from 2011 to 2021: A Systematic Review and Meta-Analysis. J. Pain Res..

[70] Li S., Feng X., Bian H. (2022). Optogenetics: Emerging strategies for neuropathic pain treatment. Front. Neurol..

[71] Iyer S.M., Vesuna S., Ramakrishnan C., Huynh K., Young S., Berndt A., Lee S.Y., Gorini C.J., Deisseroth K., Delp S.L. (2016). Optogenetic and chemogenetic strategies for sustained inhibition of pain. Sci. Rep..

[72] Sun L., Liu R., Guo F., Wen M.-Q., Ma X.-L., Li K.-Y., Sun H., Xu C.-L., Li Y.-Y., Wu M.-Y. (2020). Parabrachial nucleus circuit governs neuropathic pain-like behavior. Nat. Commun..

[73] Su P.-Y.P., Zhang L., He L., Zhao N., Guan Z. (2022). The Role of Neuro-Immune Interactions in Chronic Pain: Implications for Clinical Practice. J. Pain Res..

[74] Kalliomäki J., Attal N., Jonzon B., Bach F.W., Huizar K., Ratcliffe S., Eriksson B., Janecki M., Danilov A., Bouhassira D. (2013). A randomized, double-blind, placebo-controlled trial of a chemokine receptor 2 (CCR2) antagonist in posttraumatic neuralgia. Pain.

[75] Miller R.E., Tran P.B., Das R., Ghoreishi-Haack N., Ren D., Miller R.J., Malfait A.-M. (2012). CCR2 chemokine receptor signaling mediates pain in experimental osteoarthritis. Proc. Natl. Acad. Sci. USA.

[76] Liu S. (2023). Targeting the Chemokine Ligand 2–Chemokine Receptor 2 Axis Provides the Possibility of Immunotherapy in Chronic Pain. Eur. J. Pharmacol..

[77] Lazaridou A., Paschali M., Vilsmark E.S., Sadora J., Burton D., Bashara A., Edwards R.R. (2023). Biofeedback EMG alternative therapy for chronic low back pain (the BEAT-pain study). Digit. Health.

[78] Nahin R.L. (2022). Use of Multimodal Multidisciplinary Pain Management in the US. JAMA Netw Open..

